# A Novel Cell-Based High-Throughput Screening Model for Inhibitors Targeting Influenza Virus Hemagglutinin–α-2,6-Sialic Acid Interaction

**DOI:** 10.3390/biom16091253

**Published:** 2026-08-29

**Authors:** Keyu Guo, Xiaofang Chen, Chenyin Wang, Yaru Liu, Chao Liu, Yexiang Wu, Xiuyong Fan, Yanni Xu, Shuyi Si, Yongxin Zhang, Jing Zhang

**Affiliations:** Institute of Medicinal Biotechnology, Chinese Academy of Medical Sciences & Peking Union Medical College, Beijing 100050, China

**Keywords:** high-throughput screening model, influenza viruses, hemagglutinin, α-2,6-sialic acid, protein–protein interaction, inhibitors

## Abstract

Rising drug resistance undermines current anti-influenza virus therapies. Although targeting the hemagglutinin (HA)–sialic acid receptor interaction is a promising strategy, progress is impeded by the lack of subtype-independent screening models. Herein, we established a fluorescence-based cell high-throughput model using fluorescein isothiocyanate-conjugated Sambucus Nigra Lectin (FITC-SNA) as a stable HA surrogate and α-2,6-sialyltransferase (ST6GAL1)-overexpressing MDCK cells to mimic the HA–receptor interface. This platform was designed to serve as an efficient primary screening tool to rapidly filter large compound libraries for potential binders to the receptor-binding interface. Screening 10,000 compounds identified Obatoclax Mesylate and Ethylparaben as primary hits. Both exhibited broad-spectrum HA inhibition activity, validating the model’s capability to identify compounds interfering with viral attachment. Further cellular antiviral assays revealed cytotoxicity for both compounds, resulting in low selectivity indexes (SIs), indicating that while these molecules effectively target the interaction site, they require substantial structural optimization for therapeutic use. Molecular docking confirmed their binding to type A H1N1, H3N2, and B/Victoria HA proteins, while ADMET predictions highlighted specific structural optimization needs to mitigate toxicity. In conclusion, this subtype-independent, highly specific high-throughput screening (HTS) model provides an efficient and reliable platform for early-stage influenza drug discovery and lead compound development.

## 1. Introduction

Influenza viruses remain a major global health threat, causing annual epidemics worldwide, which infect 5–15% of the global population [1] and result in approximately 3–5 million severe cases and 290,000–650,000 respiratory deaths annually, alongside substantial economic burdens [2,3]. Historical and recent outbreaks [4,5] underscore the persistent risk of novel strains. Current control strategies face substantial limitations. Vaccines require annual reformulation due to antigenic drift/shift, with variable protective efficacy (10–60%) [6]. Meanwhile, mainstream antivirals targeting neuraminidase or M2 channels suffer from widespread resistance [7,8] and even novel polymerase inhibitors carry emerging resistance risks [9]. These challenges underscore the urgent need for novel broad-spectrum anti-influenza strategies with high resistance barriers.

Viral hemagglutinin (HA) binding to host receptors is a prerequisite for entry [10], making it an ideal therapeutic target. Disrupting this interaction prevents both viral attachment and dissemination, thereby exerting dual prophylactic and therapeutic effects. However, current screening platforms for this target have notable drawbacks. Live virus or pseudovirus entry assays are labor-intensive, time-consuming [11], and require elevated biosafety levels (BSL-2/3) [12]. Plaque reduction, microneutralization, and Hemagglutinin Inhibition (HAI) assays are low-throughput and semi-quantitative, limiting large-scale drug discovery [13]. Phage-displayed antibody libraries yield antibodies rather than small molecules [14,15]. Host-directed glycans modifications alter receptors rather than serve as direct screening platforms [16,17]. Consequently, there is a critical need for robust, high-throughput, subtype-independent models to directly assess HA–receptor binding inhibition.

To address this, we developed a novel cell-based high-throughput screening (HTS) platform using Sambucus Nigra Lectin (SNA), which exhibits high-affinity binding to α-2,6-sialylated glycoproteins [18] as a stable surrogate for diverse HA subtypes, along with MDCK cells overexpressing α-2,6-sialyltransferase (ST6GAL1). We acknowledge that while SNA does not share the full structural homology of HA, it serves as an excellent functional mimic of the receptor-binding site (RBS) for the purpose of primary screening. The primary goal of this model is to identify compounds that can competitively inhibit the binding of the probe to the α-2,6-sialic acid receptor. Given that the assay detects interference at the protein–receptor interface, the mechanism of action for identified hits is inherently diverse as compounds may exert their effects by binding directly to the viral HA-alternative probe (SNA), by occupying the host receptor, or through mixed modes of action. Consequently, while this approach enables rapid, subtype-independent filtering of large libraries without live viruses, hits generated by this surrogate strategy require rigorous secondary validation using actual HA proteins and infectious viruses to confirm true antiviral potential and exclude false positives. Herein, we describe the establishment, optimization, and validation of this model, report the identification of hit compounds, and discuss its advantages and limitations.

## 2. Materials and Methods

### 2.1. Reagents

FITC-SNA (Xi’an Qiyue, Xi’ an, China), 96-well plates and puromycin (Biosharp, Nanjing, China), polybrene (Thermo Fisher, Waltham, MA, USA), sialidase (MedChemExpress, Monmouth Junction, NJ, USA), 1% guinea pig red blood cells (GPRBC) (Nanjing SenBeiJia, Nanjing, China), 6′-Sialyllactose (6′-SL), 3′-Sialyllactose (3′-SL) and Wheat Germ Agglutinin (WGA) (Shanghai yuanye, Shanghai, China), Dimethyl sulfoxide (DMSO, Beyotime Shanghai, China), and the pCDH-CMV-ST6GAL1-EF1-Puro vector (MeisenCTCC, Guangzhou, China) were purchased from the indicated manufacturers. psPAX2 and pMD2.G were purchased from Addgene (Watertown, USA). MDCK cells were maintained in-house. A diverse, drug-like small-molecule library (10 mM stock solutions in DMSO, sourced from TargetMol (Boston, MA, USA), and Selleck (Houston, TX, USA) was provided by the Institute of Medicinal Biotechnology (IMB), Chinese Academy of Medical Sciences (CAMS) and Peking Union Medical College (PUMC).

### 2.2. Construction of a Stable MDCK Cell Line Overexpressing α-2,6-Sialyltransferase (ST6GAL1)

The ST6GAL1 gene (Gene ID: 6480, NM_173216.2) was subcloned into the pCDH-CMV-MCS-EF1-Puro lentiviral vector. Lentiviral particles produced in HEK293T cells (recombinant plasmid: psPAX2: pMD2.G at a ratio of 2:1:1) were used to transduce MDCK cells with 8 μg/mL polybrene. Following pool-level qPCR validation, single clones were sorted via flow cytometry. ST6GAL1 overexpression was confirmed by reverse transcription quantitative PCR (RT-qPCR) (forward: 5′-AACTCTCAGTTGGTTACCACAGA-3′; reverse: 5′-GGTGCAGCTTACGATAAGTCTT-3′) and Western blot (anti-ST6GAL1, 1:3000, Proteintech) with GAPDH serving as the loading control.

### 2.3. Establishment and Optimization of Cell-Based HTS Assay

Unless otherwise specified, ST6GAL1-overexpressing MDCK cells were seeded at 1 × 10^5^ cells/well in 96-well plates and cultured to 80% confluency. Following treatments, cells were washed with PBS and relative fluorescence unit (RFU) was measured (Enspire 2104 Reader, PerkinElmer, Inc. (Revvity), Waltham, MA, USA, Ex/Em: 485/535 nm). Key parameters were optimized as follows: FITC-SNA concentration: Cells were incubated with serially diluted FITC-SNA (5, 10, 20, 40, 80, and 160 μg/mL) for 60 min at 37 °C; Reaction temperature: Cells were incubated with 20 μg/mL FITC-SNA at 4 °C, 25 °C, or 37 °C for 60 min; Reaction time: Cells were incubated with 20 μg/mL FITC-SNA at 37 °C for 15, 30, 45, or 60 min; DMSO tolerance: Cells were treated with 0–10% DMSO and 20 μg/mL FITC-SNA; PBS Washing cycles: After FITC-SNA incubation, cells were washed 1, 2, or 3 times with PBS.

### 2.4. Model Validation and Specificity

Inhibitory activity was evaluated by pre-incubating cells with test compounds (6′-SL or WGA) or controls for 60 min, followed by 20 μg/mL FITC-SNA for 60 min at 25 °C. Half maximal inhibitory concentration (IC_50_) values were calculated using GraphPad Prism 9.0. Specificity was validated by comparing 200 μM 6′-SL (α-2,6-inhibitor) and 200 μM 3′-SL (α-2,3-analog). For sialidase treatment, confluent cells were incubated with 100 mU Sialidase at 37 °C for 2 h prior to washing and probing. The Z’ factor was calculated from positive (FITC-SNA) and negative (PBS) control groups.

### 2.5. Primary and Secondary Screening of Inhibitors

Cells were treated with 50 μM test compounds for 60 min at room temperature (RT), followed by FITC-SNA (20 μg/mL) for 60 min at 25 °C in the dark. Positive (20 μM 6′-SL) and negative (cells only) controls were run in triplicate. Compounds exhibiting ≥ 50% inhibition were advanced to secondary screening (3.125–200 μM) for IC_50_ determination.

### 2.6. Cell Viability Assay [19]

Cell viability was determined using Cell Counting Kit-8 (CCK-8, Promega, USA). MDCK cells were seeded at 5 × 10^3^ cells/well and incubated overnight. After 48 h incubation with compounds serially diluted two-fold (1.56, 3.125, 6.25, 12.5, 25, 50, 100, and 200 μg/mL), the original culture medium was replaced with fresh medium containing 10% CCK-8 solution. Following 3 h co-cultivation at 37 °C, absorbance at 450 nm (OD_450_) was measured using a Multimode Reader (Envision, PE, Shanghai, China). The percentage of viable cells was calculated relative to cells treated with DMSO alone (solvent control). The half-maximal cytotoxic concentration (CC_50_) values were computed using GraphPad Prism 9.0 software. The rate of cell viability was calculated as follows:

$$\mathrm{Cell}\;\mathrm{viability}\;\mathrm{rate}\;(\%)=[\frac{\left({\mathrm{OD}}_{experimental}-{\mathrm{OD}}_{blank}\right)}{\left({\mathrm{OD}}_{\mathrm{control}}-{\mathrm{OD}}_{\mathrm{blank}}\right)}]\times 100\%$$

where OD_control_ is the absorbance value of solvent control wells, OD_experimental_ is the absorbance value of compound-treated wells, and OD_blank_ is the absorbance value of wells containing only medium and CCK-8 solution.

### 2.7. HAI Assay [20]

#### 2.7.1. Determination of HA Titer

HA titers were determined using a microplate method. Serial two-fold dilutions of virus suspension were performed in PBS across wells 1–12 of a V-shaped 96-well plate, starting with 100 µL in well 1 and discarding 50 µL from the final well. Subsequently, 50 µL of 1% GPRBC was added to wells 1–12 with well 13 serving as the cell control (PBS only). The plate was mixed and incubated at RT for 45 min. Agglutination was assessed by tilting the plate: complete agglutination was identified by a diffuse cell layer, whereas non-agglutination appeared as a compact button. The highest dilution causing complete agglutination was defined as the HA titer (HAU).

#### 2.7.2. HAI Assay of Positive Compounds

Obatoclax Mesylate was serially two-fold diluted in PBS containing 1% DMSO. The diluted compounds were first incubated with A/Puerto Rico/8/1934 (H1N1) virus solution, followed by the addition of 1% GPRBC suspension. As high concentrations (100–25 μM) caused hemolysis due to toxicity, non-hemolytic concentrations (<25 μM) were selected for subsequent assays. The compound was serially diluted and incubated with B/Phuket/3073/2013 (B/Yamagata) and B/Brisbane/60/2008 (B/Victoria) virus solutions, followed by 1% GPRBC suspension. The same protocol was used for Ethylparaben, with serial two-fold dilutions incubated with A/Puerto Rico/8/1934 (H1N1), A/Aichi/2/1968 (X31) (H3N2), B/Brisbane/60/2008 (B/Victoria), and B/Phuket/3073/2013 (B/Yamagata) virus solutions.

### 2.8. Detecting the Effects of Compounds on Influenza Virus CPE [21]

#### 2.8.1. Virus Propagation

The selected influenza viruses (A/Puerto Rico/8/1934 (H1N1), A/Aichi/2/1968 (X31) (H3N2), B/Brisbane/60/2008 (B/Victoria), and B/Phuket/3073/2013 (B/Yamagata)) were propagated in 9-day-old specific pathogen-free (SPF) embryonated chicken eggs. The viruses were inoculated into the allantoic cavity and incubated at 37 °C for 48 h. The allantoic fluids were harvested, clarified by centrifugation (4 °C, 3000 rpm, 5 min), and stored at −80 °C.

#### 2.8.2. CPE

Under aseptic conditions, adherent logarithmic-phase MDCK cells were treated with serially diluted compounds (6 gradients) for 1 h, then infected with influenza virus (MOI = 0.1). After 48 h incubation in DMEM maintenance medium supplemented with 1 µg/mL TPCK-treated trypsin, CCK-8 was added for 4 h, and OD_450_ values were measured and corrected by blank control. Using GraphPad Prism, perform nonlinear regression with “log(inhibitor) vs. response–Variable slope’’ using the logarithm of compound concentration (log[C]) as the X-axis and CPE inhibition rate (%) or cell viability (%) as the Y-axis, to obtain the Median Effective Concentration (EC_50_): $\mathrm{CPE}\;\mathrm{inhibition}\;\mathrm{rate}\;(\%)=\frac{\left({\mathrm{OD}}_{\mathrm{compound}}-{\mathrm{OD}}_{\mathrm{virus}}\right)}{\left({\mathrm{OD}}_{\mathrm{cell}\;\mathrm{control}}-{\mathrm{OD}}_{\mathrm{virus}}\right)}\times 100\%$ and selectivity index (SI = CC_50_/EC_50_) values were computed. GraphPad Prism 9.0 was used for statistical analysis (x ± s, *t*-test/ANOVA, * *p* < 0.05 significant). Experiments were independently repeated three times.

### 2.9. Molecular Docking of Compounds to Influenza HA

The crystal structures of A/Puerto Rico/8/34(H1N1) HA (PDB: 9N8P) [22], A/Aichi/2/1968 (X31) (H3N2) HA (PDB: 3ZTJ) [23], and B/Brisbane/60/2008(B/Victoria) HA (PDB: 4FQM) [24] were processed using the QuickPrep module of Molecular Operating Environment (MOE) 2024 (hydrogenation, energy minimization, and non-protein molecule removal). Potential binding sites were predicted via the Site Finder module. Compound 3D structures were retrieved from PubChem, optimized via the Wash function (Amber10-EHT force field), and energy-minimized. Conformational searching was performed using the LowModeMD algorithm (10,000 cycles, RMS gradient 0.005 nm). Docking was conducted via the Dock module (Triangle Matcher placement, London dG initial scoring, GBVI/WAS refined scoring), with top 100 poses retained for analysis.

### 2.10. ADMET Prediction

ADMET properties were predicted using ADMETlab 3.0 and SwissADME. Compound structures were converted to SMILES format via ChemDraw Ultra 14.0. Key parameters (physicochemical properties, absorption, distribution, metabolism, excretion, toxicity) were analyzed to evaluate druggability.

### 2.11. Statistical Analysis

Data are presented as mean ± SD (*n* = 3). Statistical significance was determined by one-way ANOVA with Tukey’s post hoc test using GraphPad Prism 9.0, where * *p* < 0.05, ** *p* < 0.01, *** *p* < 0.001, and **** *p* < 0.0001.

## 3. Results

### 3.1. Establishment and Feasibility Validation of the Cell-Based HTS Model

**Figure 1:**
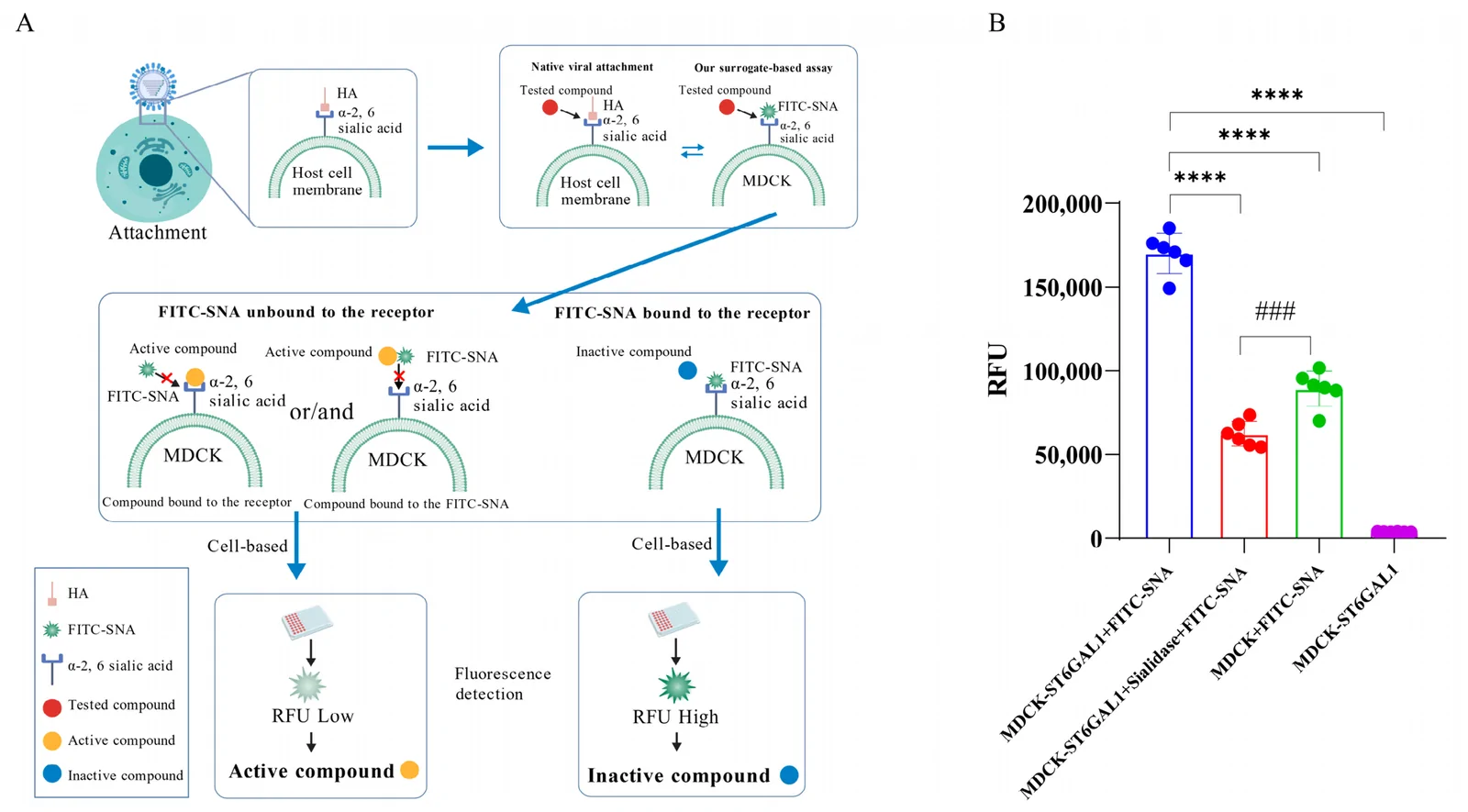
Establishment and feasibility confirmation of the fluorescence-based high-throughput screening (HTS) platform. (**A**) Illustration of the screening platform utilizing FITC-labeled Sambucus nigra lectin (SNA) as a functional HA surrogate and ST6GAL1-overexpressing MDCK cells as receptor carriers. The model detects inhibitors through relative fluorescence unit (RFU) reduction when compounds interfere with SNA-α-2,6-sialic acid binding. Created with BioGDP.com. [25] (**B**) Quantitative analysis of model feasibility validation. The bar graph compares the RFU of four distinct experimental conditions to validate the model: (i) MDCK cells with stable ST6GAL1 overexpression demonstrating elevated α-2,6-sialic acid expression; (ii) ST6GAL1-overexpressing cells pretreated with sialidase to enzymatically remove sialic acid residues, serving as a specificity control; (iii) parental wild-type MDCK cells showing baseline endogenous receptor levels; and (iv) ST6GAL1-overexpressing cells without probe addition to account for cellular autofluorescence. Significant fluorescence elevation in the ST6GAL1-overexpressing group versus wild-type confirms successful genetic modification, while the reduction following sialidase treatment confirms the specificity of FITC-SNA for its target receptor. *n* = 6; **** *p* < 0.0001, ### *p* < 0.001.

The fluorescence-based HTS model was established using FITC-labeled SNA (α-2,6-sialic acid-specific lectin) and MDCK cells stably overexpressing ST6GAL1 (Figures S1 and S2). The model quantifies SNA-α-2,6-sialic acid acceptor binding via RFU, where strong fluorescence occurs when FITC-SNA binds to cells, while inhibitory compounds reduce RFU (Figure 1A). To verify the engineered cell line and probe specificity, we compared RFU across four groups. Parental MDCK cells showed low basal fluorescence, indicating weak endogenous α-2,6-sialic acid acceptor. In contrast, ST6GAL1-overexpressing cells exhibited an approximately two-fold RFU increase, confirming elevated receptor abundance via stable transduction. Sialidase pretreatment significantly reduced binding fluorescence, verifying probe specificity, while unprobed cells showed negligible autofluorescence (Figure 1B). Together, these data validate the biological foundation and feasibility of the HTS model.

### 3.2. Systematic Optimization of the Cell-Based HTS Model

**Figure 2:**
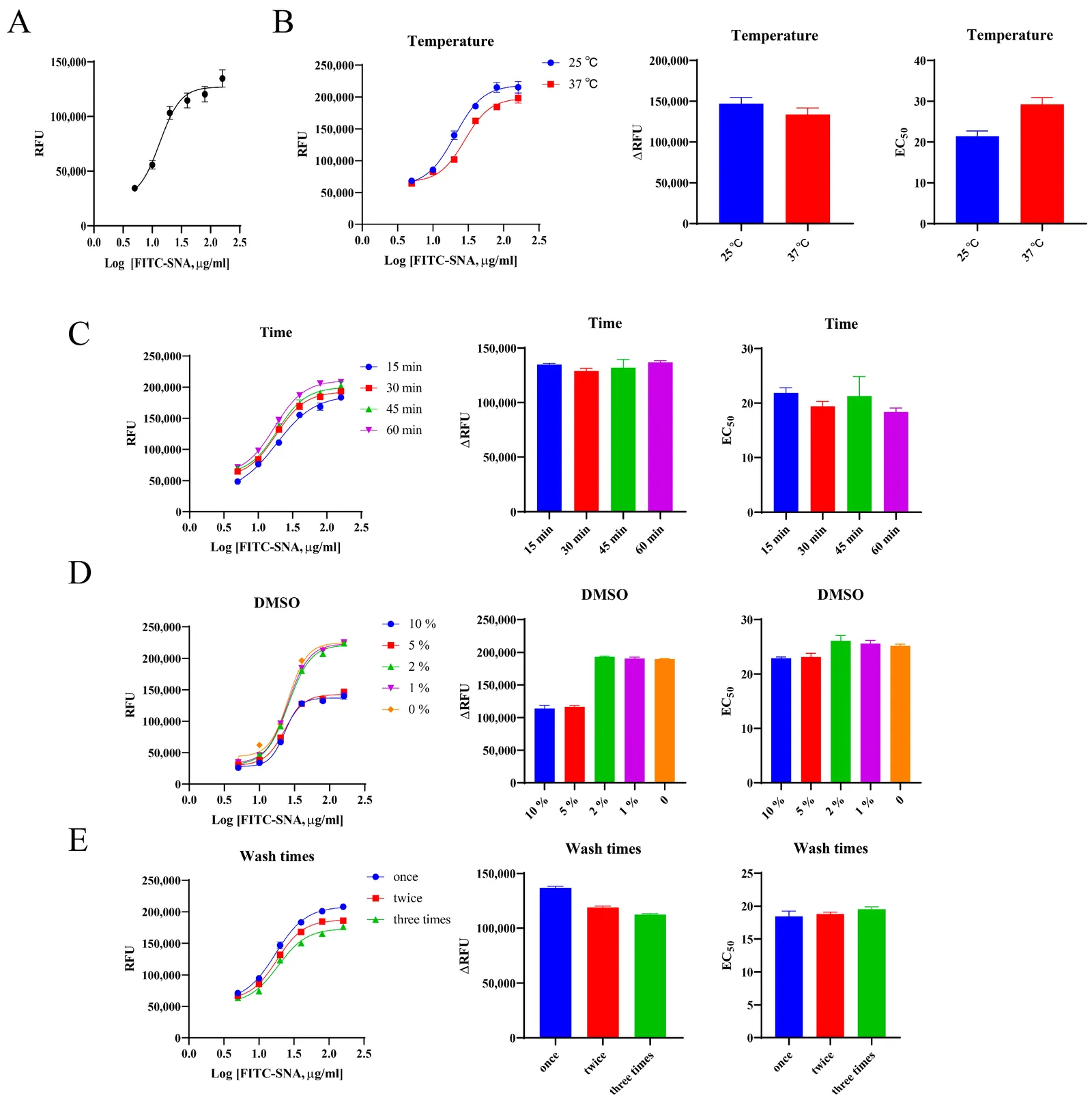
Optimization of the newly established HTS assay. (**A**) Dose–response curve assessing the effect of increasing concentrations of FITC-SNA (5 to 160 μg/mL) on RFU to determine the optimal probe concentration for saturation binding. A concentration of 20 μg/mL was selected to maintain sensitivity while minimizing background. (**B**) Comparative analysis of binding stability across different incubation temperatures (4 °C, 25 °C, and 37 °C) to identify conditions that preserve interaction integrity while ensuring experimental convenience. Given that the binding affinity remained consistent between 25 °C and 37 °C, a temperature of 25 °C was selected as the standard condition to facilitate experimental handling. (**C**) Time-course profile plotting RFU against incubation duration (15, 30, 45, and 60 min) to establish the minimum time required to reach binding equilibrium. Although the signal reached a plateau rapidly, a 60 min incubation period was defined as the optimal duration to ensure complete reaction equilibrium. (**D**) Evaluation of solvent tolerance by measuring RFU in the presence of varying concentrations of DMSO (0% to 10%) to ensure the assay can accommodate compound libraries dissolved in DMSO without signal interference. Since DMSO concentrations exceeding 2% significantly attenuated the binding signal, the final assay protocol restricted the solvent tolerance to a maximum of 2% DMSO. (**E**) Determination of the optimal number of PBS washing steps (1, 2, or 3 cycles) required to remove unbound probe effectively without disrupting cell-bound complexes. As a single wash cycle proved sufficient to eliminate unbound probe without disturbing the specific binding, one wash was established as the standard procedure. As a single wash cycle proved sufficient to eliminate unbound probe without disturbing the specific binding, one wash was established as the standard procedure. All measurements were performed in quadruplicates.

Based on the feasibility validation, key parameters were systematically optimized. RFU increased concentration-dependently with FITC-SNA levels, exhibiting a typical sigmoidal pattern and stabilizing between 20 and 40 μg/mL (Figure 2A). To conserve reagent and minimize background while maintaining sensitivity, 20 μg/mL was selected as the optimal working concentration. Temperature effects were evaluated at this fixed concentration. No significant differences in RFU, ΔRFU (calculated as the change in relative fluorescence units, representing the specific binding signal), or EC_50_ were observed between 25 °C and 37 °C (Figure 2B), indicating stable binding within this range. For operational convenience, 25 °C was chosen. Since insufficient incubation time can lead to incomplete binding and compromise inhibitor evaluation, the incubation time was optimized. RFU reached a plateau, while ΔRFU and EC_50_ remained consistent across 15, 30, 45, and 60 min (Figure 2C), indicating rapid binding saturation. To ensure reaction completeness, 60 min was selected. DMSO tolerance was assessed as it may interfere with binding activity. Concentrations ≤ 2% did not significantly alter binding curves, EC_50_, or ΔRFU, whereas 5% and 10% DMSO significantly decreased both parameters (Figure 2D). Accordingly, the working DMSO concentration was limited to ≤ 2%. Finally, to remove unbound FITC-SNA without disrupting the reaction, washing cycles were evaluated. RFU, ΔRFU, and EC_50_ showed no significant differences after 1, 2, or 3 washes (Figure 2E), indicating that a single wash effectively removes unbound probe. Systematic optimization yielded the optimal conditions as FITC-SNA: 20 μg/mL; Incubation: 60 min at 25 °C; DMSO: ≤ 2%; and PBS washes: 1 cycle.

### 3.3. Validation, Specificity, and Reliability of the Optimized Model

**Figure 3:**
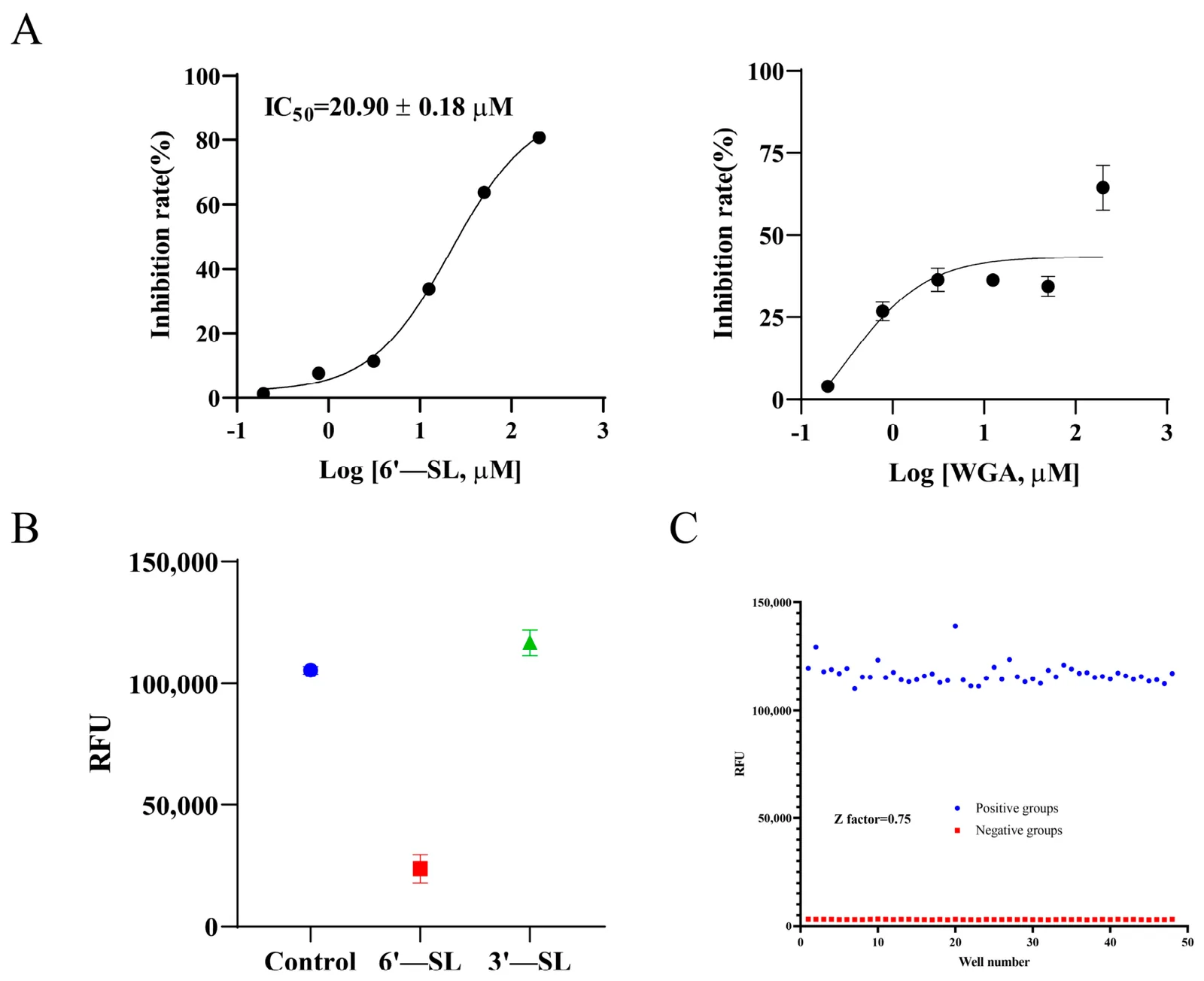
Functional validation and robustness assessment of the established screening model. (**A**) Dose–response curves demonstrating the inhibitory capacity of known binding agents on the FITC–SNA receptor interaction. 6′-Sialyllactose (6′-SL), a structural analog of α-2,6-sialic acid, and Wheat Germ Agglutinin (WGA), a lectin that binds sialic acid, were used to verify that the assay can detect competitive inhibition or steric blockade at the receptor interface. (**B**) Bar graph verifying the assay’s selectivity for the α-2,6 linkage specificity. The inhibitory effect of 200 μM 6′-SL (specific α-2,6-sialic acid analog) is contrasted with that of 200 μM 3′-sialyllactose (3′-SL, an α-2,3-sialic acid analog). The significant reduction in RFU observed with 6′-SL, but not with 3′-SL, confirms the model’s specificity for the α-2,6-sialic acid receptor configuration targeted by human influenza viruses. (**C**) Scatter plot representing the Z’-factor calculation to evaluate assay performance and suitability for HTS. The plot displays fluorescence values for the positive control (FITC-SNA only) and the negative control (PBS only). A calculated Z’-factor of 0.75 indicates excellent separation between the control groups and confirms the high reliability and reproducibility of the system. Data were obtained from triplicate experiments.

Following optimization, model efficacy was validated using 6′-SL (an α-2,6-sialic acid structural analog) and WGA (an α-2,6-sialic acid binder). Theoretically, they are capable of inhibiting SNA–receptor interaction by targeting SNA or the receptor, respectively. Consistent with this, 6′-SL dose-dependently reduced RFU, yielding an IC_50_ of 16.86 ± 1.36 μM. WGA’s weak affinity prevented IC_50_ determination despite observable binding (Figure 3A), confirming the model’s utility for screening inhibitors.

Subsequently, specificity was validated by comparing 200 μM 6′-SL (α-2,6-inhibitor) and 200 μM 3′-SL (an α-2,3-sialic acid analog). Since SNA exhibits high selectivity for α-2,6-linked sialic acid over α-2,3-isomers, 6′-SL significantly decreased RFU, whereas 3′-SL caused almost no change (Figure 3B), confirming the model’s high specificity and ability to exclude α-2,3-sialic acid-related interference.

Finally, the Z’ factor was employed to evaluate model quality and reproducibility. The established model generated a Z’ factor of 0.75 (Figure 3C), meeting the HTS standard (≥0.5) and demonstrating strong stability and high reliability [26].

### 3.4. Screening and Cytotoxicity Evaluation of Hit Compounds

**Figure 4:**
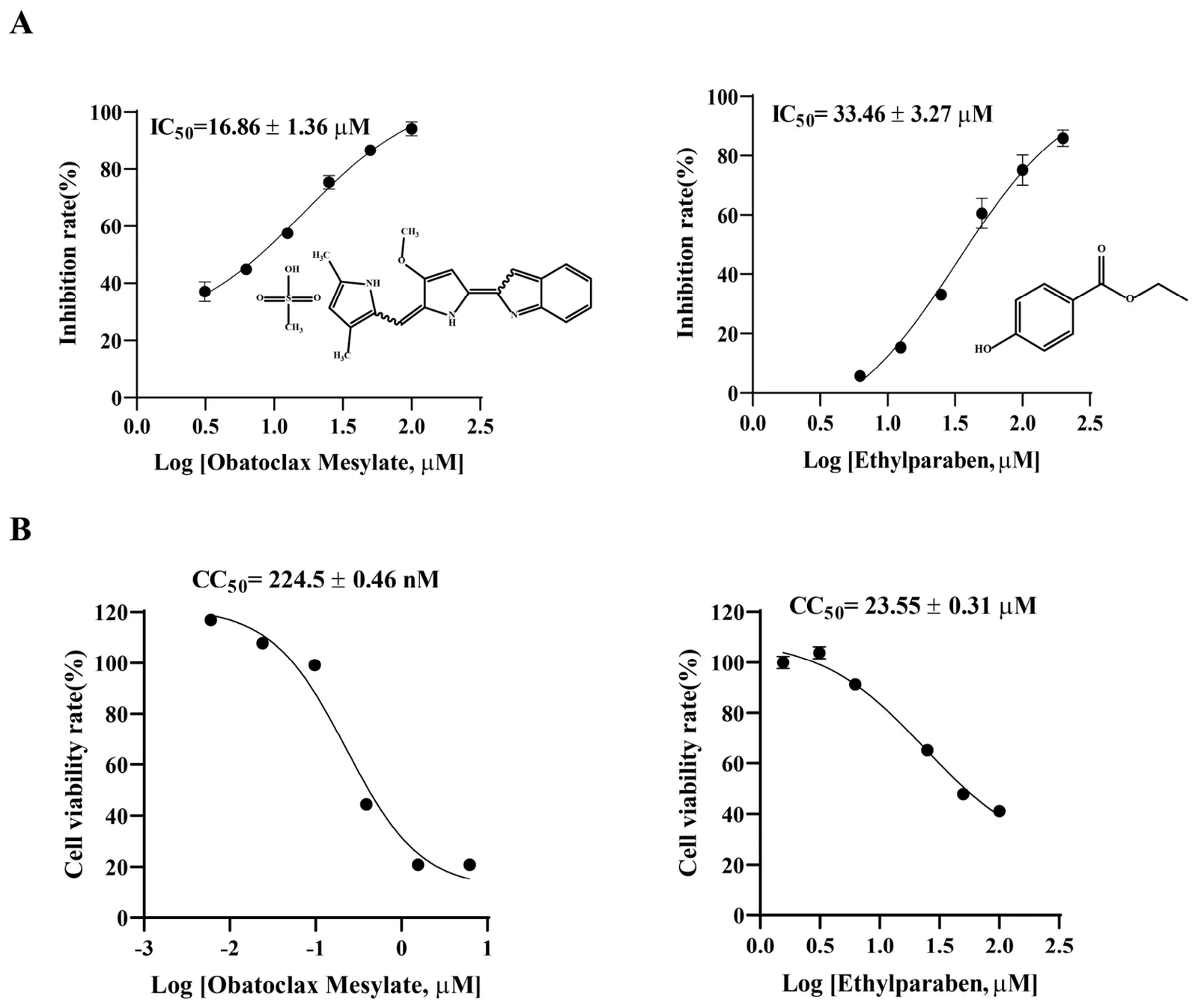
Identification and cytotoxicity profiling of primary hit compounds from screening. (**A**) Structural representation and dose-dependent inhibition profiles of the two confirmed hit compounds, Obatoclax Mesylate and Ethylparaben. The graphs illustrate the concentration-response relationships (IC_50_ determination) of these molecules in inhibiting the FITC-SNA binding signal, validating their potential as blockers of the HA–receptor interface. (**B**) Cytotoxicity assessment of the identified hits using the Cell Counting Kit-8 (CCK-8) assay. MDCK cells were exposed to a range of concentrations of Obatoclax Mesylate and Ethylparaben for 48 h to determine the 50% cytotoxic concentration (CC_50_). The curves quantify the reduction in cell viability relative to compound concentration. Results are derived from three independent experiments.

Screening 10,000 compounds (50 μM) yielded 5 primary hits (≥50% inhibition), with secondary screening confirming Obatoclax Mesylate (IC_50_ = 16.86 ± 1.36 μM) and Ethylparaben (IC_50_ = 33.46 ± 3.27 μM) as positive hits (Figure 4A). CCK-8 assays revealed high cytotoxicity for Obatoclax Mesylate (48 h CC_50_ = 224.5 ± 0.46 nM) and moderate cytotoxicity for Ethylparaben (48 h CC_50_ = 23.55 ± 0.31 μM) (Figure 4B). However, 2 h (covering the total screening time window) acute toxicity validation at the screening concentration (50 μM) confirmed > 90% cell viability for both compounds, effectively ruling out false positives from acute cell death (Figure S3).

### 3.5. HAI Assay

HAU were determined as follows, A/Puerto Rico/8/1934 (H1N1) 1:256, A/Aichi/2/1968 (X31) (H3N2) 1:128, B/Phuket/3073/2013 (B/Yamagata) 1:64, and B/Brisbane/60/2008 (B/Victoria) 1:32.

**Figure 5:**
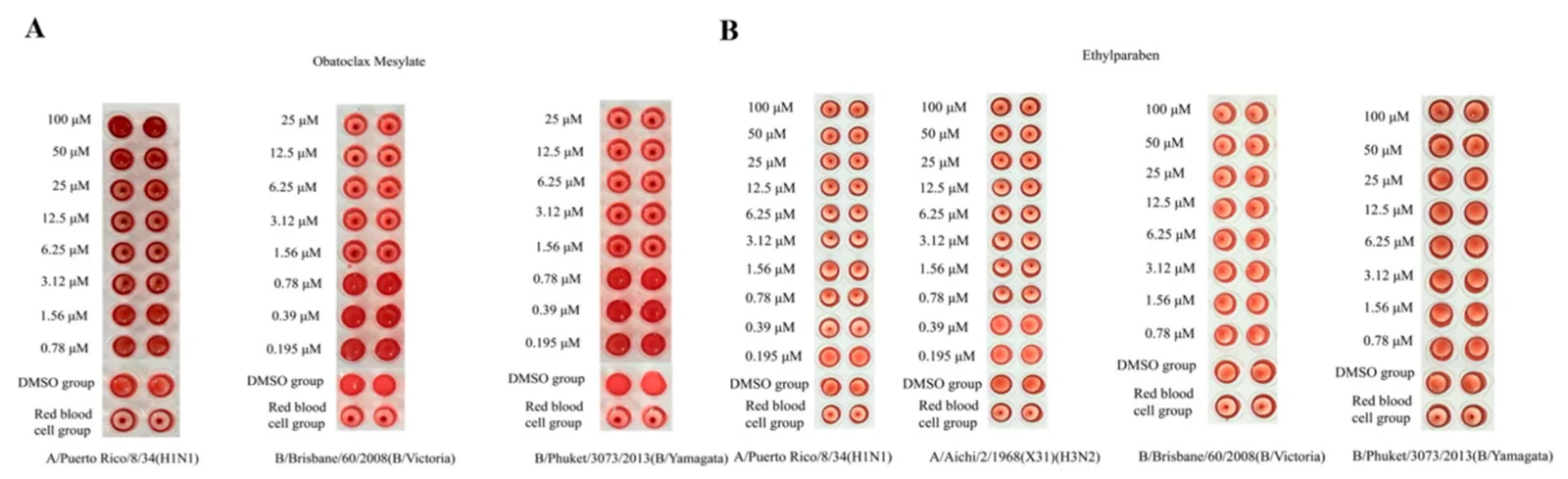
Evaluation of broad-spectrum antiviral activity using hemagglutination inhibition (HAI) assays. The capacity of hit compounds to block viral attachment was assessed against four distinct influenza virus strains using guinea pig red blood cells (GPRBCs). HAI assays were performed with 256 HA units (H1N1), 128 HA units (H3N2), 16 HA units (B Victoria), or 32 HA units (B Yamagata) of virus alongside serial dilutions of the compounds. (**A**) Visualization of HAI activity for Obatoclax Mesylate. The results demonstrate the compound’s ability to inhibit hemagglutination induced by A/Puerto Rico/8/1934 (H1N1), B/Phuket/3073/2013 (B/Yamagata), and B/Brisbane/60/2008 (B/Victoria). Sedimentation patterns confirm effective receptor binding blockade at non-hemolytic concentrations. (**B**) HAI activity of Ethylparaben against the same viral panel, including A/Aichi/2/1968 (X31) (H3N2). These data confirm that Ethylparaben targets hemagglutinin (HA) to prevent red blood cell agglutination across multiple subtypes, validating the mechanism of action identified in the primary screen.

Obatoclax Mesylate was two-fold serially diluted in PBS, and incubated with virus and 1% GPRBC suspension. Against H1N1, hemolysis (cytotoxicity) occurred at 100–25 μM, sedimentation (inhibition) at 12.5–3.12 μM, and agglutination (no inhibition) at 1.56–0.78 μM. Non-hemolytic concentrations (<25 μM) were used for subsequent HAI assays. Against B/Yamagata, sedimentation occurred at 25–1.56 μM and agglutination at 0.78–0.39 μM. Against B/Victoria, sedimentation was observed at 25–1.56 μM. These results indicate that Obatoclax Mesylate significantly inhibits HA–sialic acid receptor binding across multiple influenza serotypes (Figure 5A). Ethylparaben was evaluated similarly. Against H1N1, sedimentation occurred at 100–0.39 μM, and agglutination at <0.195 μM. Against H3N2, sedimentation occurred at 100–0.78 μM, and agglutination at <0.39 μM. Against B/Victoria, sedimentation occurred at 100–1.56 μM, and agglutination at <0.78 μM. Against B/Yamagata, the inhibitory concentration range was relatively higher, with sedimentation at 100–50 μM and agglutination at <25 μM. Collectively, Ethylparaben exhibits varying degrees of HAI activity against all four tested strains (Figure 5B).

### 3.6. CPE Inhibition Assay

The cytotoxicity observed in the CC_50_ assays necessitated a careful evaluation of antiviral efficacy. Obatoclax Mesylate was unevaluable in this assay due to its high cytotoxicity at the effective concentrations. Conversely, Ethylparaben effectively inhibited CPE across multiple strains, with EC_50_ values of 103.00 ± 0.80 μM (H1N1), 50.69 ± 9.10 μM (H3N2), 35.50 ± 0.77 μM (B/Victoria), and 41.37 ± 10.00 μM (B/Yamagata) (Table 1). Although the Selectivity Index (SI) for Ethylparaben was low (<1 across all tested strains), this does not negate the compound’s inherent antiviral activity. As demonstrated in the preceding HAI assays, Ethylparaben directly interacts with viral HA and effectively inhibits receptor binding. Therefore, the low SI values indicate that the compound possesses dual properties. It exerts a specific antiviral effect by blocking viral attachment while simultaneously exhibiting significant host cell toxicity. The narrow antiviral window suggests that the effective antiviral concentrations closely overlap with cytotoxic concentrations, making the observed cellular protection difficult to fully manifest in the CPE context. Consequently, while these molecules are not suitable for direct therapeutic application in their current form due to this toxicity, they effectively serve as tool compounds that validate the screening model’s ability to target the receptor interface. Furthermore, these findings highlight the need for further structural optimization to mitigate cytotoxicity, widen the therapeutic window, and fully unmask the potential of this attachment-inhibiting mechanism.

Cells were seeded into 96-well plates and cultured overnight, followed by inoculation with various influenza viruses at a MOI of 0.1. CC_50_ and EC_50_ were measured via the CPE inhibition method. SI values were computed as the ratio of CC_50_ to EC_50_. All experiments were conducted in triplicate (*n* = 3), and the results are expressed as mean ± SD.

### 3.7. Molecular Docking

As both compounds exhibited HAI activity against influenza A and B viruses, molecular docking was performed to investigate their interaction with the HA of H1N1, H3N2, and B/Victoria strains. Crystal structures of A/Puerto Rico/8/34 (H1N1) HA (PDB: 9N8P), A/Aichi/2/1968 (X31) (H3N2) HA (PDB: 3ZTJ), and B/Brisbane/60/2008 (B/Victoria) HA (PDB: 4FQM) were prepared using the QuickPrep module in MOE 2024 software for hydrogenation, energy minimization, and removal of non-protein molecules. Potential small-molecule binding sites were predicted using the MOE 2024 Site Finder module, identifying site3 (for 9N8P) and site2 (for 4FQM) as the primary targets. Structurally, these sites partially overlap with the canonical RBS. Key amino acids for site3 (PDB:9N8P) include GLY97, ASP98, ILE100, ILE182, HIS184, ARG211, ARG212, PHE213, THR214, GLU216, ARG229, ASN231, TYR233, LYS572, THR206, SER207, ASN208, TYR209, and ASN210. For site2 (PDB:4FQM), they include YS103, LEU110, LEU111, GLY113, TYR114, ASP183, ALA214, ASN215, GLY216, VAL217, THR218, THR219, TYR221, ASP246, TYR247, MET248, VAL249, LYS251, ARG276, SER414, GLY415, MET417, LEU420, HIS421, GLU423, ILE424, GLU419, ASN422, and GLU426. Notably, site 3 (for 9N8P) in H1N1 encompasses critical residues of the RBS 220-loop, including ARG211, ARG212, and PHE213. This partial overlap suggests that the compounds exert their inhibitory effects by partially occupying the receptor interface, likely causing steric hindrance that blocks sialic acid access rather than acting purely allosterically.

**Figure 6:**
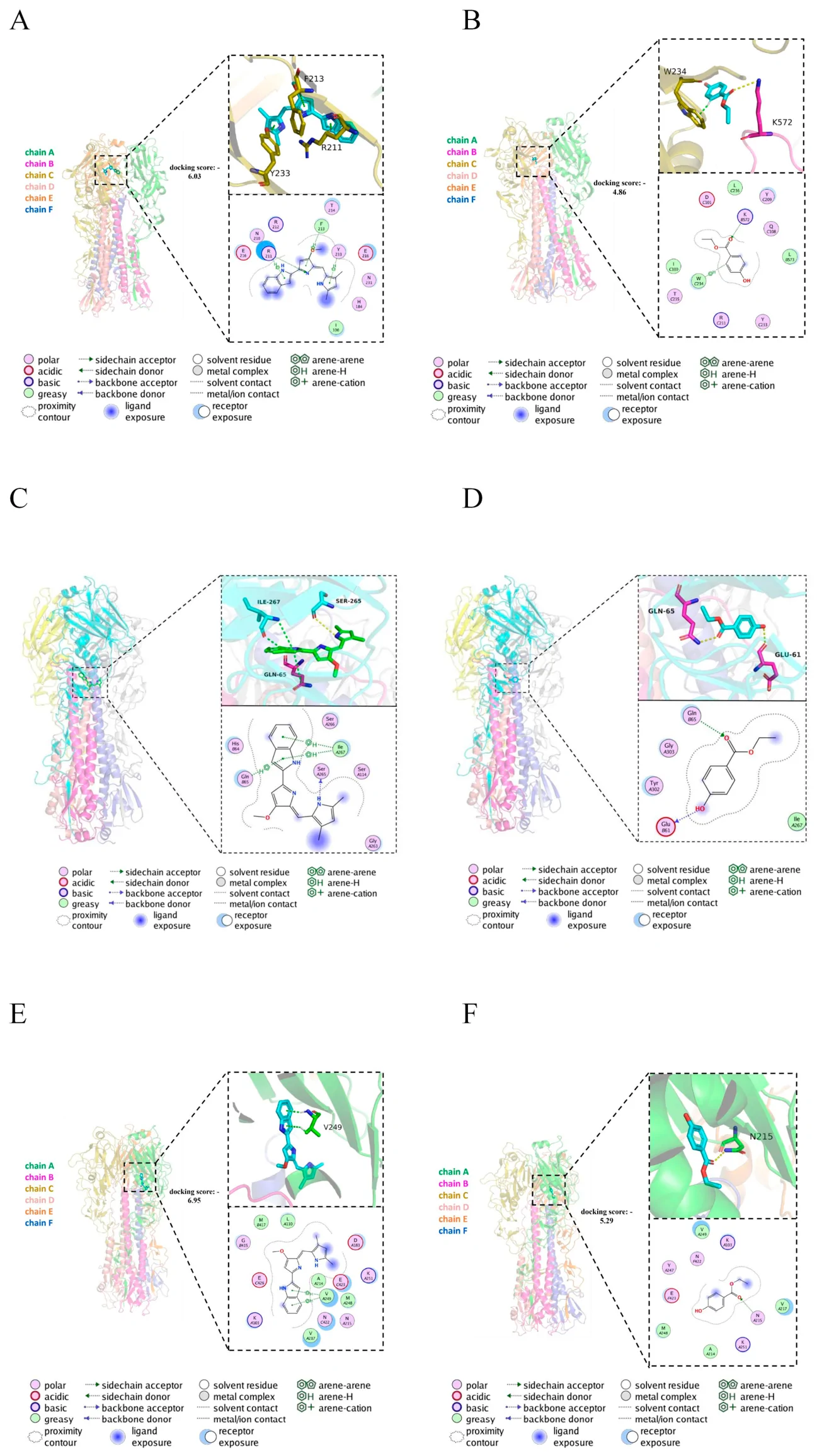
Molecular docking analysis predicting the interaction mechanisms of hit compounds with influenza Hemagglutinin. Computational simulations were performed to visualize the binding poses of Obatoclax Mesylate and Ethylparaben within the receptor-binding sites of H1N1, H3N2, and B/Victoria HA proteins. (**A**) The binding configuration of Obatoclax Mesylate docked into the HA protein of A/Puerto Rico/8/1934 (H1N1; PDB: 9N8P). The compound occupies a pocket near the head region, forming a hydrogen bond with residue ARG211 and engaging in π-H stacking interactions with PHE213, ARG211, and TYR233. (**B**) Interaction diagram of Ethylparaben complexed with H1N1 HA (9N8P). The ligand forms a hydrogen bond with LYS572 and participates in π-H stacking with TRP234. (**C**) Binding mode of Obatoclax Mesylate interacting with the HA of B/Brisbane/60/2008 (B/Victoria; PDB: 4FQM). The docking pose reveals π-H stacking interactions with VAL249. (**D**) Docking results for Ethylparaben with B/Victoria HA (4FQM), illustrating a hydrogen bond formation with ASN215. (**E**) Binding mode of Obatoclax Mesylate interacting with the HA of A/Aichi/2/1968 (X31) (H3N2; PDB: 3ZTJ). The docking pose reveals a hydrogen bond formation with residue SER265 and π-H stacking interactions with GLN65 and ILE267. (**F**) Docking results for Ethylparaben with H3N2 HA (3ZTJ), illustrating hydrogen bond formations with residues GLU61 and GLN65. In all panels, the left images depict the global positioning of the ligand within the protein structure, the top right panels highlight specific atomic interactions (hydrogen bonds in yellow, π-H stacking in green), and the bottom right panels provide two-dimensional interaction maps summarizing the binding residues and interaction types.

Binding affinity is characterized by the docking score, where lower scores indicate stronger affinity. In the H1N1 strain (9N8P), the Obatoclax Mesylate complex showed a docking score of −6.03 (Table 2), featuring a hydrogen bond with ARG211 and π-H stacking with PHE213, ARG211, and TYR233 (Figure 6A), an interaction that confirms binding to key RBS residues. Conversely, the 9N8P-Ethylparaben complex scored −4.86 (Table 2), forming a hydrogen bond with LYS572 and π-H stacking to TRP234 (Figure 6B). For the H3N2 strain (3ZTJ), the Obatoclax Mesylate complex recorded a score of −5.34 with a hydrogen bond to SER265 and π-H stacking with GLN65 and ILE267 (Figure 6C), whereas the Ethylparaben complex achieved a score of −5.24, forming hydrogen bonds with GLU61 and GLN65 (Figure 6D). Regarding the B/Victoria strain (4FQM), the Obatoclax complex (site2) showed a score of −6.95 (Table 2) featuring π-H stacking with VAL249 (Figure 6E), whereas the Ethylparaben complex (site2) scored −5.29 (Table 2), forming a hydrogen bond with ASN215 (Figure 6F). Structural analysis indicated that the H3N2 interaction residues (GLU61, GLN65, SER265, and ILE267) are located within the HA1 head domain but are situated in the periphery adjacent to the canonical RBS, rather than within the RBS itself, which is formed by the 130-loop, 150-loop, 190-helix, and 220-loop. These results collectively indicate that the antiviral activity of these compounds is mediated through their interaction with key sites at or near the receptor-binding interface of the HA head.

### 3.8. Pharmacokinetic Property Evaluation

**Figure 7:**
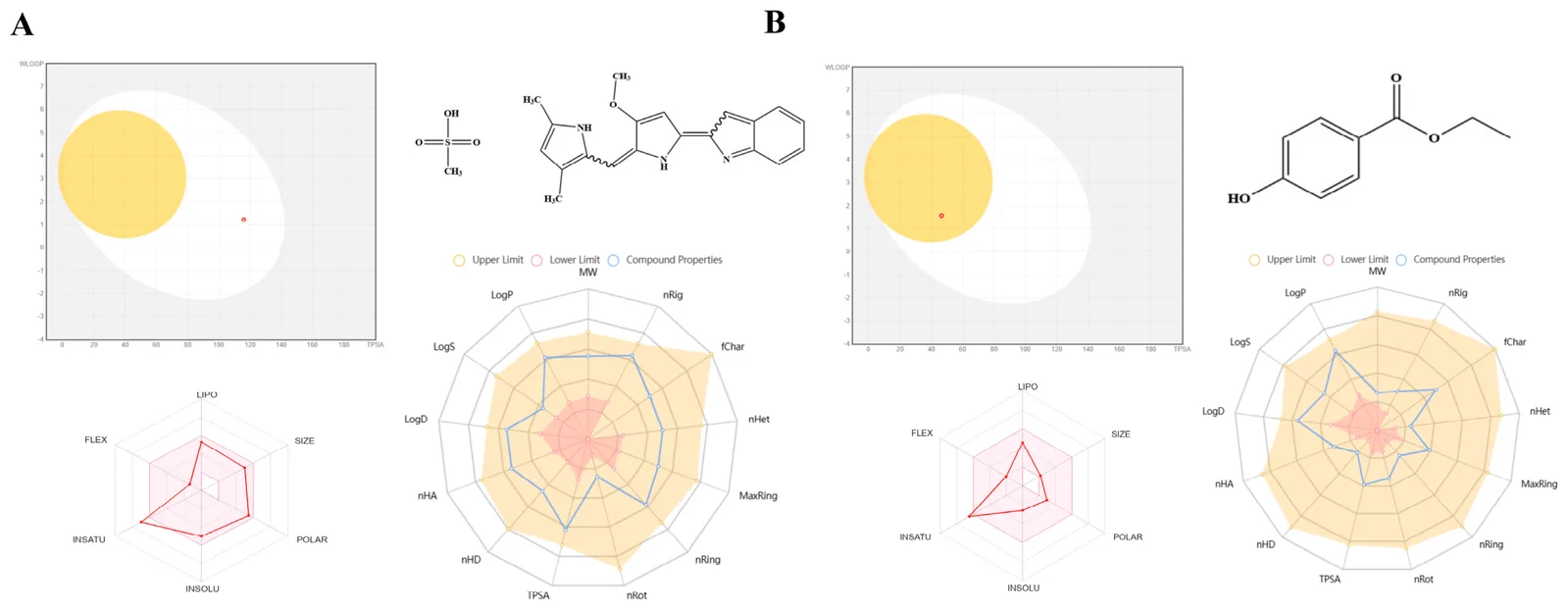
Comprehensive in silico prediction of pharmacokinetic and toxicological properties (ADMET). Computational models were utilized to assess the drug-likeness and safety profiles of the identified hit compounds to guide future structural optimization. (**A**) Pharmacokinetic profiling of Obatoclax Mesylate. The top left panel displays a BOILED-Egg model predicting gastrointestinal absorption (white region) and brain penetration (yellow region), indicating low blood–brain barrier (BBB) penetration. The lower left panel shows a SwissADME bioavailability radar comparing the molecule’s physicochemical properties (lipophilicity, polarity, etc.) against optimal ranges (pink area). The right panel presents an ADMETlab 3.0 radar, highlighting specific liabilities such as high plasma protein binding and poor permeability that need to be addressed. (**B**) Pharmacokinetic profiling of Ethylparaben. Similar to (**A**), the BOILED-Egg plot shows moderate absorption and BBB penetration potential. The SwissADME bioavailability radar confirms good compliance with drug-likeness rules (Lipinski, etc.), while the ADMETlab 3.0 radar identifies potential issues with human intestinal absorption and specific toxicity alerts (e.g., eye irritation) that require chemical modification. These visualizations provide a roadmap for medicinal chemistry efforts to improve the therapeutic index of the lead compounds.

ADMET predictions revealed distinct profiles for Obatoclax Mesylate and Ethylparaben, each with favorable and limiting properties. For Obatoclax Mesylate (Table 3, Figure 7A), the computational analysis identified several advantageous properties, including oral drug-compliant MW, logP, and logD that comply with oral drug standards, alongside high synthetic accessibility, low hERG risk, and minimal blood–brain barrier (BBB) penetration. However, its pharmacokinetic profile is significantly constrained by poor permeability and extremely high plasma protein binding (PPB). The prediction further indicated strong inhibition of CYP1A2 and CYP3A4, coupled with high toxicity risks, low clearance, and a short half-life. In contrast, Ethylparaben (Table 3, Figure 7B) demonstrated optimal physicochemical properties, exhibiting good drug-likeness, and ease of synthesis. While it satisfies Lipinski, Pfizer, and GSK rules, it fails to meet the Golden Triangle standard and presents with low Fsp3 and MCE-18 values, alongside two ALARM NMR thiol reactivity alerts. Regarding absorption, Ethylparaben shows acceptable Caco-2 and moderate MDCK permeability, yet it is predicted to have poor human intestinal absorption (HIA) and low bioavailability. Its distribution profile features moderate PPB, favorable volume of distribution, and moderate BBB penetration. Metabolically, the compound acts as a strong inhibitor of CYP1A2 and CYP2C19 and is a major substrate of CYP2C9. The excretion parameters suggest moderate clearance and a short half-life. Toxicity assessments indicate low systemic risks and no toxicophore alerts, although the compound is predicted to have high eye irritation and significant estrogen receptor activity.

## 4. Discussion

Targeting the HA–receptor interaction offers a promising strategy against escalating influenza resistance [27,28], however, current platforms are limited by high biosafety requirements, low throughput, and an inability to directly screen receptor-binding blockers. To address these gaps, we developed an SNA surrogate-based cell model. It is crucial to contextualize the biological relevance of this surrogate-based approach. While using SNA instead of native HA allows for subtype-independent screening, it inherently introduces the possibility of detecting compounds that target the receptor, the lectin probe, or even the fluorophore, rather than the viral protein itself. Consequently, this model is best conceptualized as the initial filter in a multi-tiered screening funnel. Its purpose is to efficiently narrow down vast chemical libraries to a manageable subset of compounds that demonstrate activity against the receptor-binding interface. These candidates must then undergo rigorous secondary validation against actual HA proteins and infectious viruses to confirm specificity for the viral target and to exclude mechanism-based false positives (e.g., compounds that only bind the SNA probe). The fact that our identified hits (Obatoclax Mesylate and Ethylparaben) demonstrated subsequent HAI activity confirms that the model successfully identified molecules capable of interacting with the viral attachment machinery, validating the utility of this funneling strategy. Conversely, it is imperative to acknowledge the inherent trade-off regarding false negatives resulting from structural divergence. Since SNA and influenza HA lack sequence or structural homology in their receptor-binding regions, compounds that specifically bind to the unique structural pockets of the viral HA RBS without blocking the receptor interface may fail to be detected by our surrogate model. However, we posit that this screening bias could be an advantageous strategy for discovering broad-spectrum inhibitors. The structural architecture of the HA RBS pocket varies significantly across different influenza subtypes, making subtype-specific pocket binders less likely to exhibit broad efficacy. In contrast, the host α-2,6-sialic acid receptor is structurally conserved. By utilizing SNA as a functional mimic of the receptor interface, our model prioritizes the identification of compounds that target this conserved receptor binding interface, thereby enriching the screen for potential pan-subtype attachment inhibitors rather than narrow-spectrum HA-pocket binders. This approach aligns with our primary objective of establishing a subtype-independent screening platform. This strategy of prioritizing conserved interface blockers over subtype-specific pocket binders represents a shift from traditional viral protein-targeting to a host-interface-centric screening paradigm. While traditional cell-based systems, such as HAb2 cells expressing influenza HA, have been utilized for decades as virus-free models to study viral fusion and entry [29], they present notable limitations for primary HTS. HAb2 and analogous systems rely on the expression of specific viral HA subtypes [30,31], meaning that broad-spectrum screening requires the generation and maintenance of multiple cell lines to cover different strains. In contrast, our surrogate-based strategy utilizing FITC-SNA decouples the screening process from specific viral subtypes without the need for handling live viruses, thereby overcoming the subtype-specific bottlenecks of traditional cell-based platforms. Beyond the clear advantages of biosafety and accessibility, this model represents a conceptual advancement in antiviral screening strategy. By directly quantifying the attachment step, the model offers a mechanism-specific screening tool that avoids the complexity of downstream replication cycles inherent in CPE based assays. The engineering of ST6GAL1-overexpressing cells further enhances this approach by creating a defined, high-density receptor environment, overcoming the sensitivity limitations of native cell lines for HTS applications. Additionally, while advanced biosensor technologies, such as Surface Plasmon Resonance (SPR) and Bio-Layer Interferometry (BLI) [32,33], provide highly reliable kinetic data with superior performance in secondary characterization [34], their application in primary HTS of large compound libraries is significantly constrained by low throughput, high consumable costs, and the requirement for specialized instrumentation [33]. Furthermore, similar to other cell-free glycoprotein immobilization assays, biosensor substrates often fail to replicate the native lipid bilayer context, membrane fluidity, and the natural multivalent density of receptors [35,36,37,38]. In contrast, our cell-based model preserves the native membrane environment and the multivalent nature of the HA–receptor interaction, which is crucial for identifying compounds that can function in a physiologically relevant setting. By utilizing standard microplate readers, this platform bridges the gap between simple biochemical binding assays and complex live-virus infection models. It successfully balances efficiency, cost, and biological relevance, serving as a highly accessible primary screening filter that complements, rather than replaces, high-performance biosensor validations, thereby providing a more predictive tool for early-stage drug discovery. Beyond validating the screening platform itself, investigating the interaction mechanisms of the identified hits provides further structural evidence supporting the model’s capability. Our molecular docking analyses provide structural insights into how the hit compounds engage HA. Upon closer inspection of the docking poses and the residue lists, Obatoclax Mesylate and Ethylparaben were shown to partially overlap with the canonical RBS. For H1N1 (Site 3; PDB: 9N8P), interactions involve residues such as ARG211, ARG212, and PHE213, which are integral components of the 220-loop of the RBS, a structural element critical for receptor engagement [39]. For B/Victoria (Site 2; PDB: 4FQM), binding involves residues including TYR114 and ASP183 that reside in or immediately adjacent to the structural loops forming the RBS. Therefore, the compounds target regions that partially consist of the RBS (specifically structural walls or loops) and extend into surrounding sub-sites. This binding mode suggests inhibition via partial occupancy of the receptor interface, likely causing steric hindrance or local conformational distortion of RBS loops (notably the 220-loop), thereby blocking receptor access, consistent with the potent inhibition of sialic acid binding observed in our cellular assays.

Regarding the biological relevance of the hits, we observed that both compounds exhibited cytotoxicity and low SI in cellular antiviral assays. However, it is crucial to recognize that this toxicity does not negate the compounds’ inherent antiviral capabilities. The preceding HAI assays clearly demonstrated that both Obatoclax Mesylate and Ethylparaben can directly interact with viral HA proteins and effectively block hemagglutination across multiple strains. This confirms that the compounds possess genuine, target-specific antiviral activity by disrupting the viral attachment machinery. The primary limitation of these hits in their current form is the close overlap between their antiviral effective concentrations and their cytotoxic concentrations (resulting in SI < 1), which narrows the therapeutic window and precludes their immediate use as therapeutics. Rather than diminishing the value of the screening platform, this profile reinforces the role of these molecules as effective tool compounds. These compounds confirm that the model can find true attachment inhibitors. They also point to a clear next step for medicinal chemistry. We need to structurally optimize these molecules. This means keeping the HA-binding parts intact while removing the toxic elements. These findings confirm the utility of the model as a robust primary filter, while also underscoring the necessity for subsequent optimization steps to identify safer derivatives with an improved therapeutic index.

The ADMET predictions further support the utility of these hits as starting points for medicinal chemistry optimization rather than final products. Obatoclax Mesylate requires structural modifications to overcome high cytotoxicity and poor pharmacokinetics, while Ethylparaben needs optimization to improve absorption and mitigate endocrine-disruption risks. Interestingly, Ethylparaben exhibited discrepancies between molecular docking, HAI, and CPE inhibition. Its lower CPE EC_50_ compared to effective HAI concentrations suggests potential off-target effects or intracellular interference, which aligns with the observed cytotoxicity. These observations underscore the necessity of the multi-tiered validation approach (HTS > HAI > CPE) that we employed as the HTS model identifies binders, HAI confirms the mechanism of attachment inhibition, and CPE/ADMET defines the therapeutic window and safety profile.

While this multi-tiered validation strategy has proven effective for identifying and characterizing hits, it is important to acknowledge the technical limitations of the current model configuration. Most notably, the current inability to detect α-2,3-sialic acid binding blockers, critical for avian influenza, could be addressed by co-expressing α-2,3-sialyltransferase and specific lectins (e.g., MAL-II) [40]. Additionally, replacing FITC-SNA with HRP-labeled SNA to establish an ELISA-based model could further reduce costs. Regarding the readout methodology, we selected a microplate reader-based format over flow cytometry, primarily to maximize throughput and operational simplicity for primary screening. While flow cytometry offers higher sensitivity and single-cell resolution, which we indeed utilized for clonal sorting during cell line construction, it is less amenable to the rapid, large-scale processing required for HTS due to the need for cell detachment and resuspension steps [41]. The robust Z’ factor (0.75) achieved in this study confirms that the plate reader format provided sufficient sensitivity and reproducibility for the primary screen, validating it as the optimal choice for balancing efficiency and data quality. Nevertheless, this model successfully balances efficiency, cost, and specificity, thereby providing a vital tool for accelerating novel influenza therapeutics development.

## 5. Conclusions

In this study, we established and validated a fluorescence-dependent cell-based HTS model targeting the influenza HA–α-2,6-sialic acid receptor interaction. Using FITC-SNA as a subtype-independent surrogate probe and ST6GAL1-overexpressing MDCK cells, the optimized model exhibited excellent specificity and reliability, fully meeting HTS criteria. Screening 10,000 compounds identified two hits: Obatoclax Mesylate and Ethylparaben, which confirmed the model’s ability to detect inhibitors of the HA-receptor interaction. Although these compounds demonstrated broad-spectrum HAI activity, their subsequent characterization revealed significant cytotoxicity and low selectivity, precluding direct clinical application. However, the molecular docking and ADMET analyses provided a structural basis for their activity and highlighted clear directions for structural optimization to reduce toxicity. This study demonstrates that while targeting the conserved receptor interface is a viable broad-spectrum strategy, it requires careful optimization to separate antiviral efficacy from host toxicity. The subtype-independent screening platform developed here provides a robust, virus-free tool for the early-stage discovery of such inhibitors and offers a flexible foundation for future efforts to identify safer, more effective antivirals. Ultimately, this approach may facilitate a more rapid response to emerging influenza strains with pandemic potential by decoupling the initial screening step from the constraints of specific viral subtypes.

## Figures and Tables

**Table 1:** Anti-influenza CPE activity of Ethylparaben in MDCK cells.

	A/Puerto Rico/8/1934 (H1N1)	A/Aichi/2/1968 (X31) (H3N2)	B/Brisbane/60/2008 (B/Victoria)	B/Phuket/3073/2013 (B/Yamagata)
EC_50_ (μM)	103.00 ± 0.80	50.69 ± 9.10	35.50 ± 0.77	41.37 ± 10.00
SI	0.23	0.46	0.66	0.57

**Table 2:** Docking scores of compounds with influenza HA.

Docking Score	A/Puerto Rico/8/1934 (H1N1)	A/Aichi/2/1968 (X31) (H3N2)	B/Brisbane/60/2008(B/Victoria)
Obatoclax Mesylate	−6.03	−5.34	−6.95
Ethylparaben	−4.86	−5.24	−5.29

**Table 3:** Key predicted ADMET parameters for Obatoclax Mesylate and Ethylparaben.

Parameter Category	Parameter Name	Optimal Range	Obatoclax Mesylate	Ethylparaben
Physicochemical Properties	Molecular Weight (MW)	100–600	413.14	166.06
	Topological Polar Surface Area (TPSA)	0–140	107.54	46.53
	Log of Octanol/Water Partition Coefficient (logP)	0–3	2.269	2.376
	Log of Aqueous Solubility (logS)	−4–0.5 log mol/L	−3.006	−2.158
Medicinal Chemistry	Quantitative Estimate of Drug-likeness (QED)	Attractive: >0.67; Unattractive: 0.49–0.67	0.533 (Unattractive)	0.678 (Attractive)
	Fsp3 (sp^3^ Hybridized Carbons/Total Carbon Count)	≥0.42	0.19	0.222
	Synthetic Accessibility Score (SAscore)	<6 (Easy to synthesize)	3.194	1.405
	Lipinski Rule	Accepted (≤1 parameter out of range)	Accepted	Accepted
Absorption	Caco-2 Permeability	>−5.15 Log unit	−5.799	−4.375
	MDCK Permeability	Low: <2 × 10^−6^; Medium: 2–20 × 10^−6^; High: >20 × 10^−6^ cm/s	5 × 10^−6^ cm/s (Medium)	2.7 × 10^−5^ cm/s (Medium)
	Human Intestinal Absorption Probability (HIA)	Category 0 (HIA ≥ 30%: <0.5; Category 1 (HIA < 30%: ≥0.5))	0.148 (Category 0)	0.006 (Category 0)
Distribution	Plasma Protein Binding (PPB)	< 90%	98.95%	89.28%
	Fraction Unbound in Plasma (Fu)	Low: <5%; Middle: 5–20%; High: >20%	1.077% (Low)	8.521% (Middle)
Metabolism	CYP1A2 Inhibitor Probability	Category 1 (Inhibitor: ≥0.5; Category 0 (Non-inhibitor: <0.5))	0.971 (Category 1)	0.973 (Category 1)
	CYP3A4 Inhibitor Probability	Category 1 (Inhibitor: ≥0.5; Category 0 (Non-inhibitor: <0.5))	0.804 (Category 1)	0.078 (Category 0)
Toxicity	Human Hepatotoxicity (H-HT) Probability	Category 1 (Toxic: ≥0.5; Category 0 (Non-toxic: <0.5))	0.972 (Category 1)	0.014 (Category 0)
	Drug-Induced Liver Injury (DILI) Probability	Category 1 (High Risk: ≥0.5; Category 0 (No Risk: <0.5))	0.849 (Category 1)	0.161 (Category 0)
	Carcinogenicity Probability	Category 1 (Carcinogen: ≥0.5; Category 0 (Non-carcinogen: <0.5))	0.823 (Category 1)	0.212 (Category 0)
	Eye Irritation Probability	Category 1 (Irritant: ≥0.5; Category 0 (Non-irritant: <0.5))	0.062 (Category 0)	0.991 (Category 1)

## Data Availability

All data are available within the manuscript.

## Supplementary Materials

The following supporting information can be downloaded at: https://www.mdpi.com/article/10.3390/biom16091253/s1, Figure S1: Construction and validation of MDCK cell lines stably overexpressing ST6GAL1; Figure S2: Full, unprocessed, and uncropped raw blot images for Western blot analysis; Figure S3: Acute cytotoxicity assessment of Obatoclax Mesylate and Ethylparaben under screening conditions.

## References

[1] WHO This Winter’s Flu Season Epidemic Has Started—What We Know So Far and What Needs to Be Done to Control It. https://www.who.int/europe/news/item/25-12-2021-this-winter-s-flu-season-epidemic-has-started-what-we-know-so-far-and-what-needs-to-be-done-to-control-it.

[2] WHO The Burden of Influenza. https://www.who.int/news-room/feature-stories/detail/the-burden-of-influenza.

[3] WHO Influenza (Seasonal). https://www.who.int/news-room/fact-sheets/detail/influenza-(seasonal).

[4] Harrington W.N., Kackos C.M., Webby R.J. (2021). The evolution and future of influenza pandemic preparedness. Exp. Mol. Med..

[5] Mena-Vasquez J., Marco-Fuertes A., Culhane M., Torremorell M. (2025). Emerging threats of HPAI H5N1 clade 2.3.4.4b in swine: Knowledge gaps and the imperative for a One Health approach. Front. Vet. Sci..

[6] Mokalla V.R., Gundarapu S., Kaushik R.S., Rajput M., Tummala H. (2025). Influenza Vaccines: Current Status, Adjuvant Strategies, and Efficacy. Vaccines.

[7] Hussain S., Meijer A., Govorkova E.A., Dapat C., Gubareva L.V., Barr I.G., Brown S.K., Daniels R.S., Fujisaki S., Galiano M. (2025). Global update on the susceptibilities of influenza viruses to neuraminidase inhibitors and the cap-dependent endonuclease inhibitor baloxavir, 2020–2023. Antivir. Res..

[8] Batool S., Chokkakula S., Song M.S. (2023). Influenza Treatment: Limitations of Antiviral Therapy and Advantages of Drug Combination Therapy. Microorganisms.

[9] Takashita E. (2021). Influenza Polymerase Inhibitors: Mechanisms of Action and Resistance. Cold Spring Harb. Perspect. Med..

[10] Hermoso-Pinilla F.J., Valdivia A., Camarasa M.-J., Ginex T., Luque F.J. (2024). Influenza A virus hemagglutinin: From classical fusion inhibitors to proteolysis targeting chimera-based strategies in antiviral drug discovery. Explor. Drug Sci..

[11] Praditya D.F., Waluyo D., Nozaki T. (2025). Reporter-expressing viruses for antiviral drug discovery research. Front. Cell. Infect. Microbiol..

[12] Denani C.B., Setatino B.P., Pereira D., Horbach I.S., Azevedo A.S., Coutinho G., Ferroco C.L., Xavier J., Leite R., Santos E. (2025). Pseudovirus-Based Neutralization Assays as Customizable and Scalable Tools for Serological Surveillance and Immune Profiling. Pathogens.

[13] Vincent T.S., Zhu M., Parekh A., Patel U., Cloney-Clark S., Klindworth A., Silva D., Gorinson A., Miranda K., Wang M. (2024). A Modified Novel Validated High-Throughput Hemagglutinin Inhibition Assay Using Recombinant Virus-like Particles and Human Red Blood Cells for the Objective Evaluation of Recombinant Hemagglutinin Nanoparticle Seasonal Influenza Vaccine. Microorganisms.

[14] Chen I.C., Chiu Y.K., Yu C.M., Lee C.C., Tung C.P., Tsou Y.L., Huang Y.J., Lin C.L., Chen H.S., Wang A.H. (2017). High throughput discovery of influenza virus neutralizing antibodies from phage-displayed synthetic antibody libraries. Sci. Rep..

[15] Ledsgaard L., Ljungars A., Rimbault C., Sørensen C.V., Tulika T., Wade J., Wouters Y., McCafferty J., Laustsen A.H. (2022). Advances in antibody phage display technology. Drug Discov. Today.

[16] Trimarco J.D., Heaton N.S. (2022). From high-throughput to therapeutic: Host-directed interventions against influenza viruses. Curr. Opin. Virol..

[17] Trimarco J.D., Nelson S.L., Chaparian R.R., Wells A.I., Murray N.B., Azadi P., Coyne C.B., Heaton N.S. (2022). Cellular glycan modification by B3GAT1 broadly restricts influenza virus infection. Nat. Commun..

[18] Nakayama M., Itoh Y. (2022). Lectin Staining to Detect Human and Avian Influenza Virus Receptors in the Airway of Nonhuman Primates. Methods Mol. Biol..

[19] Fan J., Schiemer T., Vaska A., Jahed V., Klavins K. (2024). Cell via Cell Viability Assay Changes Cellular Metabolic Characteristics by Intervening with Glycolysis and Pentose Phosphate Pathway. Chem. Res. Toxicol..

[20] Motaghi S., Pullenayegum E., Morgan R.L., Loeb M. (2024). The role of influenza Hemagglutination-Inhibition antibody as a vaccine mediator in children. Vaccine.

[21] Li W., Wu Z., Xia Y., Tan J., Zhao H., Chen S., Li Y., Tang H., Wang G., Zhang Y. (2022). Antiviral and Antioxidant Components from the Fruits of Illicium verum Hook.f. (Chinese Star Anise). J. Agric. Food Chem..

[22] Huang Q.J., Kim R., Song K., Grigorieff N., Munro J.B., Schiffer C.A., Somasundaran M. (2025). Virion-associated influenza hemagglutinin clusters upon sialic acid binding visualized by cryoelectron tomography. Proc. Natl. Acad. Sci. USA.

[23] Corti D., Voss J., Gamblin S.J., Codoni G., Macagno A., Jarrossay D., Vachieri S.G., Pinna D., Minola A., Vanzetta F. (2011). A neutralizing antibody selected from plasma cells that binds to group 1 and group 2 influenza A hemagglutinins. Science.

[24] Dreyfus C., Laursen N.S., Kwaks T., Zuijdgeest D., Khayat R., Ekiert D.C., Lee J.H., Metlagel Z., Bujny M.V., Jongeneelen M. (2012). Highly conserved protective epitopes on influenza B viruses. Science.

[25] Jiang S., Li H., Zhang L., Mu W., Zhang Y., Chen T., Wu J., Tang H., Zheng S., Liu Y. (2025). Generic Diagramming Platform (GDP): A comprehensive database of high-quality biomedical graphics. Nucleic Acids Res..

[26] He S., Meister D., Khan S., Mohammadzadeh A., Delgadillo Silva L., Rutter G.A., Trant J.F., Lim G.E. (2025). A high-throughput screening approach to discover potential colorectal cancer chemotherapeutics: Repurposing drugs to identify novel disruptors of 14-3-3 proteins. Cell Death Dis..

[27] Smyk J.M., Szydłowska N., Szulc W., Majewska A. (2022). Evolution of Influenza Viruses-Drug Resistance, Treatment Options, and Prospects. Int. J. Mol. Sci..

[28] Maurer D.P., Vu M., Ferreira Ramos A.S., Dugan H.L., Khalife P., Geoghegan J.C., Walker L.M., Bajic G., Schmidt A.G. (2025). Conserved sites on the influenza H1 and H3 hemagglutinin recognized by human antibodies. Sci. Adv..

[29] Danieli T., Pelletier S.L., Henis Y.I., White J.M. (1996). Membrane fusion mediated by the influenza virus hemagglutinin requires the concerted action of at least three hemagglutinin trimers. J. Cell Biol..

[30] Galloway S.E., Reed M.L., Russell C.J., Steinhauer D.A. (2013). Influenza HA subtypes demonstrate divergent phenotypes for cleavage activation and pH of fusion: Implications for host range and adaptation. PLoS Pathog..

[31] Wen Z., Wu C., Chen W., Zeng X., Shi J., Ge J., Chen H., Bu Z. (2015). Establishment of MDCK Stable Cell Lines Expressing TMPRSS2 and MSPL and Their Applications in Propagating Influenza Vaccine Viruses in Absence of Exogenous Trypsin. Biotechnol. Res. Int..

[32] Serafin B., Kamen A., de Crescenzo G., Henry O. (2024). Antibody-independent surface plasmon resonance assays for influenza vaccine quality control. Appl. Microbiol. Biotechnol..

[33] Murali S., Rustandi R.R., Zheng X., Payne A., Shang L. (2022). Applications of Surface Plasmon Resonance and Biolayer Interferometry for Virus-Ligand Binding. Viruses.

[34] Holdgate G., Embrey K., Milbradt A., Davies G. (2019). Biophysical methods in early drug discovery. Admet Dmpk.

[35] Nakagawa F., Kikkawa M., Chen S., Miyashita Y., Hamaguchi-Suzuki N., Shibuya M., Yamashita S., Nagase L., Yasuda S., Shiroishi M. (2023). Anti-nanodisc antibodies specifically capture nanodiscs and facilitate molecular interaction kinetics studies for membrane protein. Sci. Rep..

[36] Patching S.G. (2014). Surface plasmon resonance spectroscopy for characterisation of membrane protein-ligand interactions and its potential for drug discovery. Biochim. Biophys. Acta.

[37] Di Iorio D., Verheijden M.L., van der Vries E., Jonkheijm P., Huskens J. (2019). Weak Multivalent Binding of Influenza Hemagglutinin Nanoparticles at a Sialoglycan-Functionalized Supported Lipid Bilayer. ACS Nano.

[38] Sieben C., Sezgin E., Eggeling C., Manley S. (2020). Influenza A viruses use multivalent sialic acid clusters for cell binding and receptor activation. PLoS Pathog..

[39] Xu R., McBride R., Paulson J.C., Basler C.F., Wilson I.A. (2010). Structure, receptor binding, and antigenicity of influenza virus hemagglutinins from the 1957 H2N2 pandemic. J. Virol..

[40] Zhu X., Nakano S., Rao V.N., Miranda H.A., Spurrier M.A., Skavicus S., Luo Z., Heaton N.S. (2026). FUT1- and GAL3ST2-mediated cellular glycan remodeling broadly restricts sialic acid-dependent viral infections. Proc. Natl. Acad. Sci. USA.

[41] Ding M., Baker D. (2021). Recent advances in high-throughput flow cytometry for drug discovery. Expert Opin. Drug Discov..

