# Supplementary material for: A Novel Cell-Based High-Throughput Screening Model for Inhibitors Targeting Influenza Virus Hemagglutinin–α-2,6-Sialic Acid Interaction
Source: Biomolecules. 2026 Aug 29;16(9):1253. doi: 10.3390/biom16091253 (PMC13604265; doi:10.3390/biom16091253)
Supplement: Supplementary file 1 [file biomolecules-16-01253-s001.zip › biomolecules-4444544-supplementary.pdf]

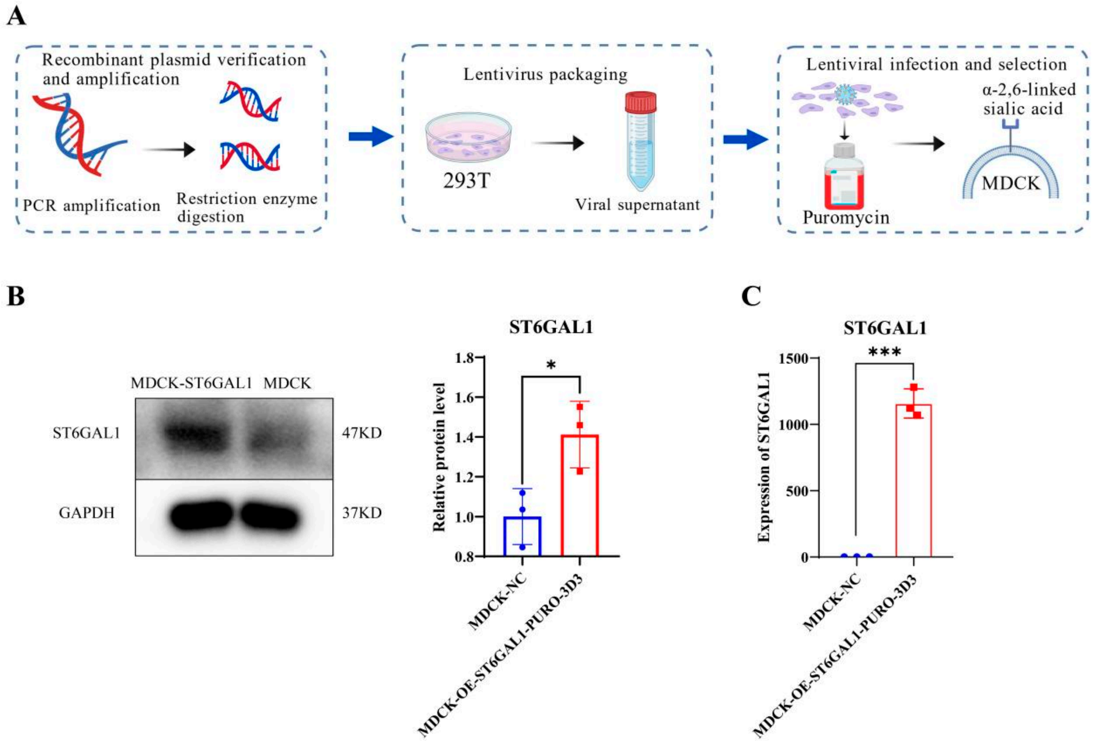

Figure S1. Construction and validation of MDCK cell lines stably overexpressing ST6GAL1. (**A**) Schematic representation of the workflow used to generate stable cell lines. The human ST6GAL1 gene (NM_173216.2) was subcloned into the lentiviral vector pCDH-CMV-MCS-EF1-Puro. Lentiviral particles were produced in HEK293T cells using a packaging system (psPAX2:pMD2.G at a ratio of 2:1:1) and subsequently used to transduce MDCK cells in the presence of polybrene (8 μg/mL). Following transduction, cells were subjected to puromycin selection, and single clones were isolated via flow cytometry. (**B**) Western blot analysis confirming the overexpression of ST6GAL1 at the protein level. Lysates from parental wild-type MDCK cells and the ST6GAL1-overexpressing clone were probed with an anti-ST6GAL1 primary antibody (1:3000). GAPDH served as the loading control. A distinct band corresponding to the molecular weight of ST6GAL1 is evident only in the transduced cells. (**C**) Quantitative real-time PCR (qPCR) analysis confirming the overexpression of ST6GAL1 at the transcriptional level. The relative mRNA expression levels were calculated using the 2-ΔΔCt method, with GAPDH as the endogenous control. The data show a significant increase in ST6GAL1 transcript levels in the engineered cell line compared to the parental control (Data presented as mean ± SD, *n*=3).

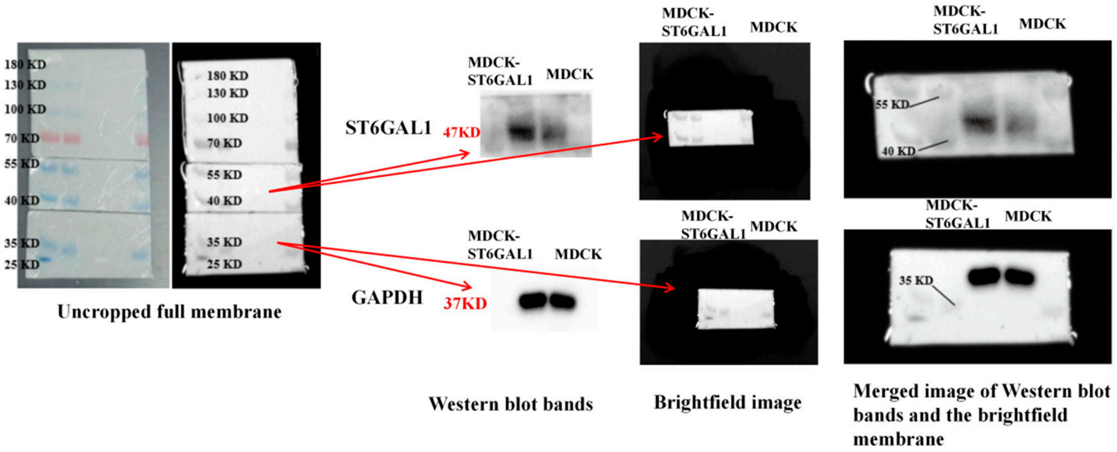

Figure S2. Full, unprocessed, and uncropped raw blot images for Western blot analysis. This figure presents the original, unedited scans of the Western blot membranes corresponding to Figure S1(B). The blot was probed sequentially with anti-ST6GAL1 and anti-GAPDH antibodies. Molecular weight markers (kDa) are indicated on the left side of the image. The full-image data confirms the specificity of the target bands and the absence of non-specific binding artifacts, in accordance with the journal's integrity guidelines.

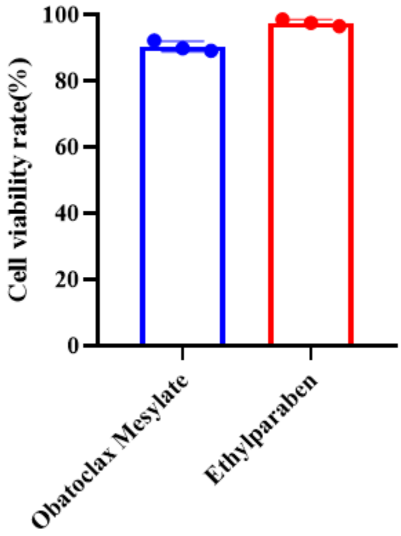

Figure S3. Acute cytotoxicity assessment of Obatoclax Mesylate and Ethylparaben under screening conditions. To rule out false-positive results in the primary screen caused by acute cell death, engineered MDCK cells (ST6GAL1-overexpressing) were treated with 50 μM of Obatoclax Mesylate or Ethylparaben. The incubation duration was set to 2 h, which precisely mimics the total exposure time in the high-throughput screening (HTS) protocol (60 min pre-incubation with compound + 60 min incubation with FITC-SNA). Cell viability was subsequently measured using the CCK-8 assay. The results demonstrate that both compounds maintained cell viability exceeding 90% at the screening concentration, confirming that the reduction in fluorescence signal observed in the primary screen was due to specific interference with the SNA-receptor interaction rather than non-specific cytotoxicity. Data are presented as mean ± SD (n = 3).
